# Altered Expression of ACE2 and Co-receptors of SARS-CoV-2 in the Gut Mucosa of the SIV Model of HIV/AIDS

**DOI:** 10.3389/fmicb.2022.879152

**Published:** 2022-04-14

**Authors:** Shuang Hu, Elise Buser, Juan Arredondo, Dylan Relyea, Clarissa Santos Rocha, Satya Dandekar

**Affiliations:** Department of Medical Microbiology and Immunology, School of Medicine, University of California, Davis, Davis, CA, United States

**Keywords:** HIV, SIV, ACE2, SARS-CoV-2, gut mucosa, co-receptors, inflammation, metabolism

## Abstract

The severe acute respiratory syndrome coronavirus 2 (SARS-CoV-2) infection, the cause of the COVID-19 pandemic, is initiated by its binding to the ACE2 receptor and other co-receptors on mucosal epithelial cells. Variable outcomes of the infection and disease severity can be influenced by pre-existing risk factors. Human immunodeficiency virus (HIV), the cause of AIDS, targets the gut mucosal immune system and impairs epithelial barriers and mucosal immunity. We sought to determine the impact and mechanisms of pre-existing HIV infection increasing mucosal vulnerability to SARS-CoV-2 infection and disease. We investigated changes in the expression of ACE2 and other SARS-CoV-2 receptors and related pathways in virally inflamed gut by using the SIV infected rhesus macaque model of HIV/AIDS. Immunohistochemical analysis showed sustained/enhanced ACE2 expression in the gut epithelium of SIV infected animals compared to uninfected controls. Gut mucosal transcriptomic analysis demonstrated enhanced expression of host factors that support SARS-CoV-2 entry, replication, and infection. Metabolomic analysis of gut luminal contents revealed the impact of SIV infection as demonstrated by impaired mitochondrial function and decreased immune response, which render the host more vulnerable to other pathogens. In summary, SIV infection resulted in sustained or increased ACE2 expression in an inflamed and immune-impaired gut mucosal microenvironment. Collectively, these mucosal changes increase the susceptibility to SARS-CoV-2 infection and disease severity and result in ineffective viral clearance. Our study highlights the use of the SIV model of AIDS to fill the knowledge gap of the enteric mechanisms of co-infections as risk factors for poor disease outcomes, generation of new viral variants and immune escape in COVID-19.

## Introduction

Severe acute respiratory syndrome coronavirus 2 (SARS-CoV-2) infection, the cause of the COVID-19 pandemic, has resulted in colossal loss of human life and global economic crisis (Wölfel et al., 2020; Hu B. et al., 2021). The virus infects and impairs lung tissue and causes severe respiratory illness (Hu B. et al., 2021). The virus attaches to the lung epithelial cells through binding of the viral spike protein with the angiotensin converting enzyme 2 (ACE2) receptor (Trypsteen et al., 2020). The level of ACE2 expression determines susceptibility to SARS-CoV-2 infection (Devaux et al., 2020; Suryamohan et al., 2021). The importance of ACE2 in SARS-CoV-2 infection is evident from the current vaccines that are targeted against viral spike protein (Krammer, 2020). The viral entry is facilitated by other host factors, including the transmembrane serine protease (TMPRSS; Mollica et al., 2020) and Cathepsin L (Yang et al., 2021), which cleave the SARS-CoV-2 spike protein for viral fusion and entry. In addition to the lung, SARS-CoV-2 susceptible cellular targets are also located in various tissues including the gastrointestinal tract and liver (Lamers et al., 2020; Salamanna et al., 2020; Wang et al., 2020; Hu S. et al., 2021; Suárez-Fariñas et al., 2021). Current anti-SARS-CoV-2 vaccines target the viral spike protein which interacts with ACE2 and initiates the viral infection of the cells (Dai and Gao, 2021). Although the virus can effectively infect cells by binding to ACE2, the clinical outcomes are highly variable among infected individuals and mechanisms underlying this variation are not fully understood (Zhang X. et al., 2020).

People with pre-existing conditions including diabetes, obesity, cardiovascular disease, age-related immune complications, or asthma have experienced severe disease due to SARS-CoV-2 infection (Apicella et al., 2020; Zhang Y. et al., 2020; Lim et al., 2021; Rosano et al., 2021). It has been recognized that increased number of people living with HIV (PLWH) were co-infected with SARS-CoV-2 and experienced worse COVID-related outcomes and increased hospitalizations compared to persons living without diagnosed HIV (Al-Harthi et al., 2021; Ambrosioni et al., 2021; Dauby and Martin, 2021; Kanwugu and Adadi, 2021; Tesoriero et al., 2021). Increased mortality rate in PLWH was attributed to HIV-induced immune dysfunction and potentially insufficient anti-SARS-CoV-2 immune response and viral clearance (Kanwugu and Adadi, 2021). Additionally, people exposed to co-infection with influenza A and SARS-CoV-2 experienced increased severity of disease (Alosaimi et al., 2021). Collectively, these findings highlight the importance of understanding the impact of pre-existing viral infections on the exposure and outcomes of SARS-CoV-2 infection. HIV, the cause of AIDS pandemic, induces CD4+ T cell depletion and immune dysfunction. The gut lymphoid tissue is an early target organ of HIV infection and dissemination (Thompson et al., 2017). Massive loss of CD4+ T cell loss and epithelial barrier disruption occur in the gut during the primary stages of HIV infection and is sustained throughout the infection. The gut mucosal damage is not readily reversed by the start of anti-retroviral therapy (ART) and the mucosal restoration is delayed compared to the peripheral blood compartment (Guadalupe et al., 2003; Veazey, 2019; Al-Harthi et al., 2021; Ambrosioni et al., 2021; Dauby and Martin, 2021; Kanwugu and Adadi, 2021). Incomplete immune recovery and residual viral replication have been documented in many HIV infected individuals despite the ART (Gaardbo et al., 2012; Reeves et al., 2018). ACE2 is highly expressed in the gut epithelium, which makes these cells susceptible to SARS-CoV-2 (Viana et al., 2020; Penninger et al., 2021). Importantly, both SARS-CoV-2 and HIV exploit the same host factors and pathways for virion assembly and release (Caillet et al., 2011; Daniloski et al., 2021), as well as for envelope incorporation for viral assembly (Qi et al., 2013; Baggen et al., 2021). Modulation of ACE2 expression is observed in the intestine of HIV-positive people receiving ART and patients with Crohn’s disease or ulcerative colitis (Fardoos et al., 2021; Suárez-Fariñas et al., 2021). Therefore, understanding the impact of HIV on the expression of SARS-CoV-2 receptors and host factors promoting the viral replication cycle is crucial in identifying the targets for effective viral clearance and especially, to determine the viral mechanisms independent of ART effects.

Metabolic dysregulation impacting amino acid and lipid metabolism is well identified in HIV infection (Ahmed et al., 2018). HIV-positive individuals receiving ART experience dyslipidemia and altered fat distribution (Ablan et al., 2006; Rasheed et al., 2008; Maggi et al., 2017). Mitochondrial dysfunction contributing to epithelial and neuronal cell impairment in HIV infection may involve multiple mechanisms including acylcarnitine and sphingomyelin dysregulation (Scarpellini et al., 2016). Changes in the tryptophan metabolism in HIV infection and production of its catabolites has a direct impact on the balance of CD4+ Th17 and Treg T cell distribution for immunomodulation (Yan et al., 2010). In addition, altered serotonin levels contribute to neurological complications in HIV-positive individuals (Gostner et al., 2015). It is important to examine whether pre-existing metabolic complications in HIV infection can impact the time course and outcomes of SARS-CoV-2 infection by complementing or accentuating COVID-19 disease and systemic inflammation (Casari et al., 2021).

We utilized the preclinical simian immunodeficiency virus (SIV) infected non-human primate model of AIDS to investigate the effect of HIV infection on the expression of SARS-CoV-2 receptor/co-receptors and molecular and metabolic networks in the gut mucosa that can support the viral infection and replication (Chandrashekar et al., 2020; Munster et al., 2020). We tested the hypothesis that HIV/SIV induced changes in the gut epithelial and immune cells increase susceptibility of the mucosal microenvironment to SARS-CoV-2 infection. We determined changes in the expression ACE2, other host factors, and functional networks by a combination of immunohistochemical, transcriptomic and metabolomic analyses. We found sustained or enhanced expression of the ACE2 receptor and host cellular factors which collectively promote viral infection in SIV infected gut compared to uninfected controls. Changes in the tryptophan and fatty acid metabolism in SIV infected gut were implicated in suboptimal immune response and energy metabolism. Thus, SIV induced gut mucosal changes can predispose the host to increased susceptibility to SARS-CoV-2 infection and disease severity.

## Materials and Methods

### Animals, Viral Infections, and Sample Collection

Specimens from 27 rhesus macaques (ranging from 4 to 13 years old) utilized in the present study were housed at the California National Primate Research Center (CNPRC). Animals were maintained in accordance with American Association for Accreditation of Laboratory Animal Care guidelines and Animal Welfare Act/Guide. The study was performed in accordance with the recommendations of the PHS (Public Health Services Policy on Humane Care and Use of Laboratory Animals). All procedures were performed according to a protocol approved by the Institutional Animal Care and Use Committee of the University of California, Davis.

For SIV infection study, 10 rhesus macaques were intravenously challenged with 1,000 TCID_50_ of SIVmac251 (Hirao et al., 2014; Crakes et al., 2019). At 7–10 weeks post infection, animals were euthanized, and tissue samples were collected. Longitudinal peripheral blood samples were collected prior to and following SIV infection to monitor viral loads and CD4+ T cell depletion. For the SARS-CoV-2 infection study, eight animals were infected intranasally with the virus (1.2×10^6^ PFU/ml) and ileum tissues were collected at animal necropsies at 14 days post infection as previously described (Shaan Lakshmanappa et al., 2021). In addition, samples from nine SIV-negative and SARS-CoV-2-negative healthy animals were included for analyses to serve as controls.

### Flow Cytometric Analysis

Peripheral blood mononuclear cells were isolated from peripheral blood samples as previously described (Crakes et al., 2019). Changes in the distribution of T cell subsets and CD4+ T cell depletion were assessed in SIV infected animals compared to uninfected controls. Multi-color immunophenotyping was performed on BD Fortessa with a minimum of 300,000 events collected. Fluorophore conjugated antibodies were obtained from BD Biosciences and used for the detection of CD45RA (MEM-56)–PE-TexasRed, CD3 (SP34)-allophycocyanin (APC)-Cy7, CD4 (OKT4)-Pacific Blue and CD8 (3B5)-APC-Cy5.5. Cells were evaluated using BD Fortessa flow cytometer. Data was analyzed using FlowJo software (v10.4.1; Tree Star, Inc., Ashland, OR) using double discrimination and live amine dyes (Invitrogen).

### Measurement of SIV Viral Loads

SIV RNA loads in plasma samples were measured by real-time reverse transcription-PCR (RT-qPCR) assay as previously described (Verhoeven et al., 2008). Briefly, viral RNA was extracted from plasma samples (Zymo Research Quick-RNA viral kit) and from ileum tissue (QIAGEN RNeasy RNA isolation kit) and reverse-transcribed to cDNA using Superscript III with random hexamer primers (ThermoFisher kit). SIVgag sequences were quantitated using an Applied Biosystems ViiA 7 detection system. The data were analyzed with ViiA RUO software (Applied Biosystem) and were extrapolated against a standard curve and RNA copies/mL or viral RNA copies/ μ g were calculated and presented.

### Immunohistochemical Analysis

Ileum tissue samples were fixed in 4% paraformaldehyde and paraffin-embedded. Tissue sections (5 μm) were deparaffinized in xylene with subsequent rehydration in 100% EtOH, 95% EtOH, and 70% EtOH (Deacon Labs). Tissue sections were permeabilized by incubation for 20 min in PBS (Gibco) containing 0.1% Triton-X100 (Sigma) then antigen retrieval through a 30 min incubation in target retrieval buffer (ACD) at 100^o^C. Slides were washed three times with PBS, then blocked for 1 h at room temperature in a PBS solution containing 15% goat serum (Sigma) and 1% FCR blocking reagent (Miltenyi Biotec). Primary antibody rabbit anti ACE2 (Invitrogen) and goat anti ZO-1 (Invitrogen) was diluted at a 1:200 concentration in blocking solution and added to tissue for overnight incubation. Tissue sections were washed 3 times for 15 min each in PBS + 0.1% Tween-20 (Biorad) and incubated for 2 h in secondary antibody Alexa 488 goat anti rabbit (Invitrogen) and Alexa 555 goat anti mouse (Invitrogen) diluted at 1:200 concentration in blocking buffer. Sections were stained with DAPI (Sigma-Aldrich), washed in PBS plus 0.1% Tween 20, then mounted with Antifade mounting media (Life Technologies Corporation) and advanced to microscopic examination.

### Confocal Microscopy and Image Analysis

Using confocal microscopy (Leica TCS SP8 STED 3X), images were acquired at optical resolution settings (20X and 63X). Three to five randomly selected regions per each tissue section were imaged, and 20× images were used for quantitative analysis. Before analysis, images underwent background subtraction in ImageJ using a rolling ball radius equal to 50. The epithelial brush border of five randomly selected villi were outlined as regions of interest (ROIs) and mean fluorescence intensity (MFI) was calculated for each ROI. An average intensity for each animal was determined as the average MFI of all villi analyzed.

### RNAseq Analysis and Gene Expression Analysis

RNAseq analysis was performed in Illumina HiSeq 4,000 system as previously described (Weber et al., 2021). In brief, total RNA was extracted using the (QIAGEN RNeasy RNA isolation kit) from ileum tissues and treated with DNase (QIAGEN RNase-Free DNase Kit). RNA integrity of all samples was above the minimum 6.0 requirement score. Library preparation for multiplexed sequencing was performed using the QuantSeq FWD kit (Lexogen) according to the manufacturer’s recommendations. Microcapillary gel electrophoresis using LabChip GX system (PerkinElmer) was used to determine fragment size distributions, followed by library quantification using a Qubit fluorometer (Invitrogen). Sequencing was performed using an Illumina HiSeq 400 system. Sequencing output was processed using Trimmomatic (0.36) to remove low-quality bases, fragments of length of <50 bp, and adapter sequences. Reads were aligned to the *Macaca mulatta* genome and quantified using the software STAR (v2.7.3a). Gene expression was visualized using heatmaps generated in GraphPad (version 9.2.0).

### Global Metabolic Profiling

Intestinal luminal contents were analyzed for changes in the metabolomic profiles and the assays were performed by Metabolon Inc. and data processed using the LIMS system as previously described (Crakes et al., 2019). Controls for analysis included a pooled matrix sample, extracted water samples, and multiple QC standards. The mean relative standard deviation of the standards was used to determine instrument variability, and experimental samples were interspersed randomly with QC samples across the platform run. Metabolite set enrichment analysis (MSEA) was performed using Metaboanalyst software. Differences in metabolite levels between groups were determined using a two-tailed Mann–Whitney test with FDR correction, and significant difference between groups was defined as *p* ≤ 0.05 and a FDR corrected value of *p* (*q*-value) ≤ 0.2. The top 25 significantly differentially detected metabolites in either direction were used as input for metabolite set enrichment analysis (MSEA) analysis. Partial least squares discriminant analysis and MSEA were peformed in metaboanalyst. Twenty-five percent of samples were filtered based on interquartile range and values were base-ten log transformed before proceeding with analysis.

### Statistical Analyses

Data represent the mean ± SEM, calculated using all data points from at least three independent experiments. Statistical significance was determined using nonparametric Mann–Whitney U test for samples with only two groups. *p* < 0.05 was considered as significant. All analysis was performed using graphpad prism (version 9.2.0).

## Results

### ACE2 Is Expressed Predominantly in the Gut Villus Epithelium

**Figure 1:**
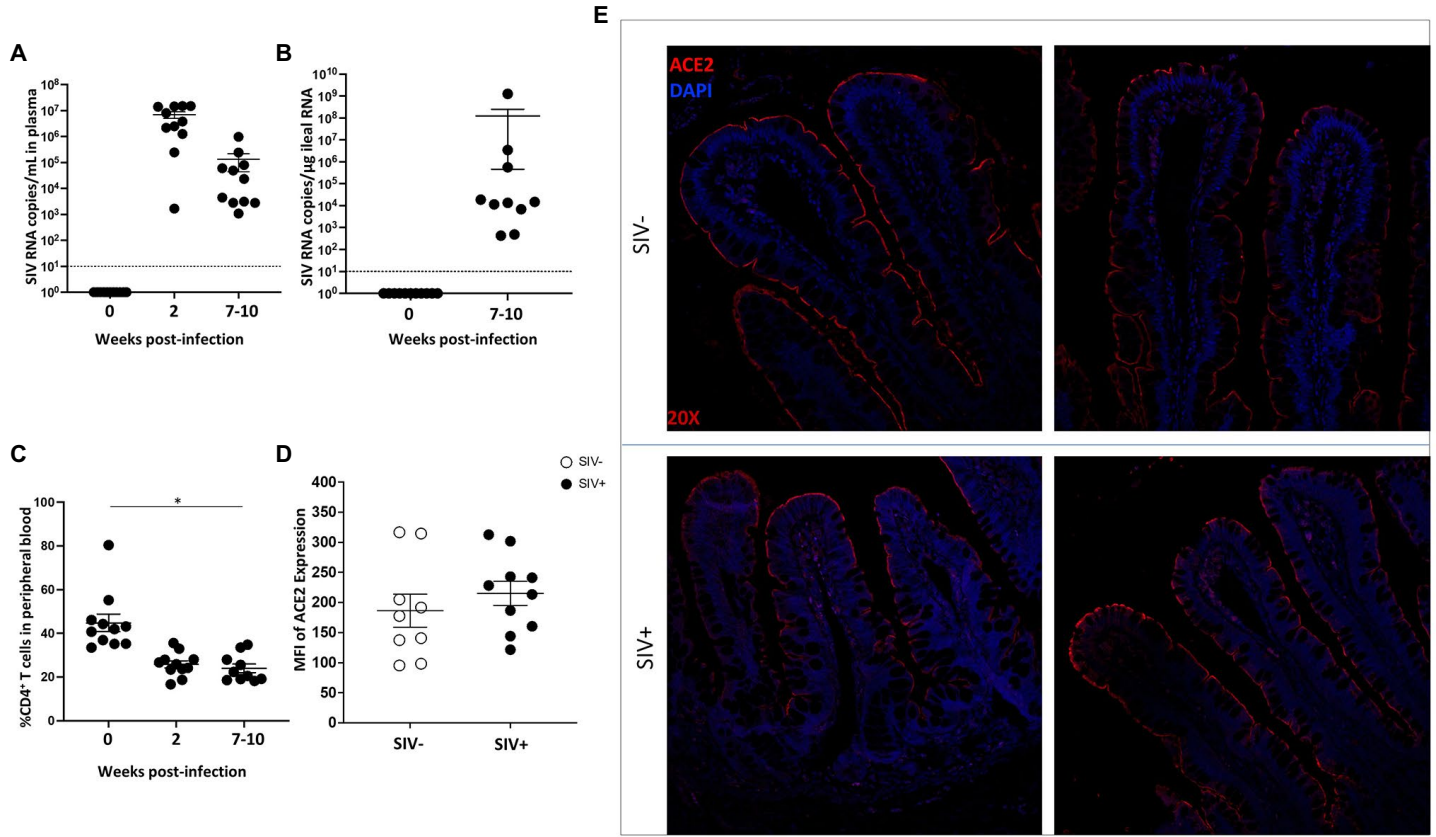
ACE2 expression is abundant in the gut epithelium of rhesus macaque in SIV infection. **(A)** and **(B)** Viral RNA copies were measured in plasma samples and in ileum tissue from SIV infected rhesus macaques (SIV+, *n* = 10). **(C)** Longitudinal CD4+ T cell depletion in peripheral blood of SIV infected animals was assessed (SIV+, *n* = 10). **(D)** Semi-quantification of ACE2 expression in intestinal villi of uninfected (*n* = 9) and SIV infected (*n* = 10) macaques by immunohistochemical analysis. **(E)** Representative images of ACE2 expression (red) in villus epithelium of uninfected and SIV infected ileum tissues by fluorescent immunostaining. Cells are identified using nuclear DNA (DAPI) staining and visualized by the blue color. Magnification: 20×. *p < 0.05.

ACE2 is known to be expressed in human intestine including the ileum and colon and is localized to the gut epithelium (Suárez-Fariñas et al., 2021). The impact of HIV infection on the expression of ACE2 can influence the outcomes of other co-infections including SARS-CoV-2 in the gut. Evaluation of ACE2 expression in HIV infected individuals in the absence of anti-retroviral therapy (ART) as compared to uninfected healthy controls will determine the effects of HIV infection independent of ART effects. The SIV model of AIDS has been well established to investigate HIV-induced enteropathy and immunodeficiency (Brenchley and Douek, 2008). We sought to determine the level of ACE2 expression and localization in the gut of SIV infected rhesus macaques. Animals were infected with SIV for 7–10 weeks and ileum tissue and peripheral blood samples were collected (Guadalupe et al., 2003). High levels of SIV RNA were detected in plasma samples (Figure 1A) as well as ileum tissue (Figure 1B). Viral infection resulted in the depletion of CD4+ T cells in the gut mucosa and peripheral blood (Figure 1C). Immunohistochemical analysis of ACE2 expression showed that ACE2 was predominantly localized to the villus epithelial cells in the gut tissue of uninfected healthy controls (Figures 1D,E). The robust level of ACE2 expression in epithelial cells was maintained in the gut of SIV infected animals. Although not statistically significant, there was a trend of increased ACE2 protein expression in the gut epithelium during SIV infection. These findings were further supported by the quantitative analysis of ACE2 fluorescent intensity in the villus epithelial cells (Figures 1D,E).

### Localization of ACE2 Expression in the Gut Remained Unaltered During SIV Infection

**Figure 2:**
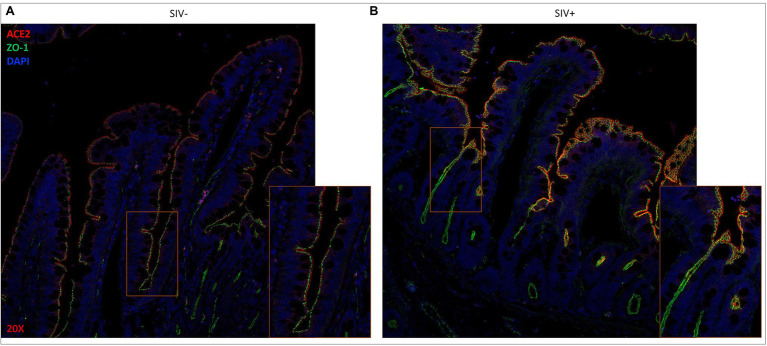
ACE2 expression remains localized along the gut epithelium in SIV infected rhesus macaque. Representative images of **(A)** uninfected and **(B)** SIV infected ileum tissues after fluorescent staining showing co-localization of ACE2 (red) and ZO-1 (green) in extracellular linings of villus epithelium. Cellular DNA (DAPI) is shown in blue to display the nuclei. Magnification: 20×.

We previously evaluated gut villus epithelial barrier changes in HIV and SIV infections by using immunohistochemical analysis of the expression and localization of ZO-1, a tight junction protein (Hirao et al., 2014; Crakes et al., 2019). In the current study, we investigated whether SIV infection led to changes in the localization and distribution of ACE2 expression in the gut microenvironment. Combined immunohistochemical analysis was performed to determine the expression and localization of ACE2 and ZO-1during SIV infection. The presence of ZO-1 expression helped the identification of the gut epithelium. The co-localization of ACE2 and ZO-1 proteins was detected in the villus epithelial lining (Figure 2). The highest expression of ACE2 was consistently seen in the differentiated epithelial cells at the villus tip and was at low to undetectable levels at the bottom of villi and in crypts. The ZO-1 expression was robust along the villus length and identified the gut epithelial cells (Figure 2). There was a trend of increased ACE2 expression in the villus epithelial lining of SIV infected gut (Figure 2).

To examine the impact of SARS-CoV-2 infection on ACE2 expression in the gut, we performed a retrospective analysis of gut tissues from SARS-CoV-2 infected rhesus macaques for ACE2 and ZO-1 expression. The experimental SARS-CoV-2 infection of rhesus macaques at UC Davis resulted in lung infection but none of the animals developed acute respiratory distress (Shaan Lakshmanappa et al., 2021). Histopathological lesions of the lungs confirmed multifocal to locally extensive interstitial pneumonia of mild to moderate severity in infected animals. Several other reports on experimental SARS-CoV-2 infection of non-human primates have also reported the occurrence of mild clinical symptoms (McMahan et al., 2021; Salguero et al., 2021). Our examination of ileum tissues of infected animals did not detect any remarkable histopathological changes compared to uninfected healthy controls.

**Figure 3:**
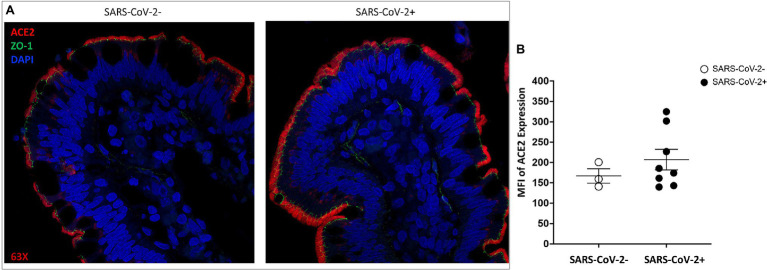
ACE2 expression is retained in the gut epithelium of SARS-CoV-2 infected rhesus macaque. Representative images of ACE2 expression ACE2 (red) and ZO-1 (green) in villus epithelium of **(A)** uninfected and SARS-CoV-2 infected ileum tissues by fluorescent immunostaining. Cell nuclei are identified by DNA staining (DAPI) in blue. Magnification: 63x. **(B)** Mean fluorescence intensity (MFI) semi-quantification of ACE2 expression levels in ileum tissues of uninfected (*n* = 3) and SARS-CoV-2 infected (*n* = 8) rhesus macaques.

We found that ACE2 expression remained robust in the gut epithelial lining and was high at the tip of the villus enterocytes in animals at 14 days post-SARS-CoV-2 infection as compared to uninfected controls (Figure 3A). A general trend of increased ACE2 expression was found in infected animals and these findings were confirmed by semi-quantitative analysis of ACE2 mean fluorescent intensity values (Figure 3B). The presence of ZO-1 expression identified the gut epithelium and confirmed the expression of ACE2 in this region with the highest expression in enterocytes (Figure 3A). SARS-CoV-2 infected animals showed a trend of increased ACE2 expression in the gut epithelium (Figure 3A) and this finding was confirmed by semi-quantification of mean fluorescent intensity values (Figure 3B). Thus, ACE2 expression in the gut epithelium was well-maintained in SARS-CoV-2 infected animals and may have increased expression compared to uninfected controls.

### SIV Infection Resulted in Activation of Gut Mucosal Gene Expression Capable of Supporting SARS-CoV-2 Replication

**Figure 4:**
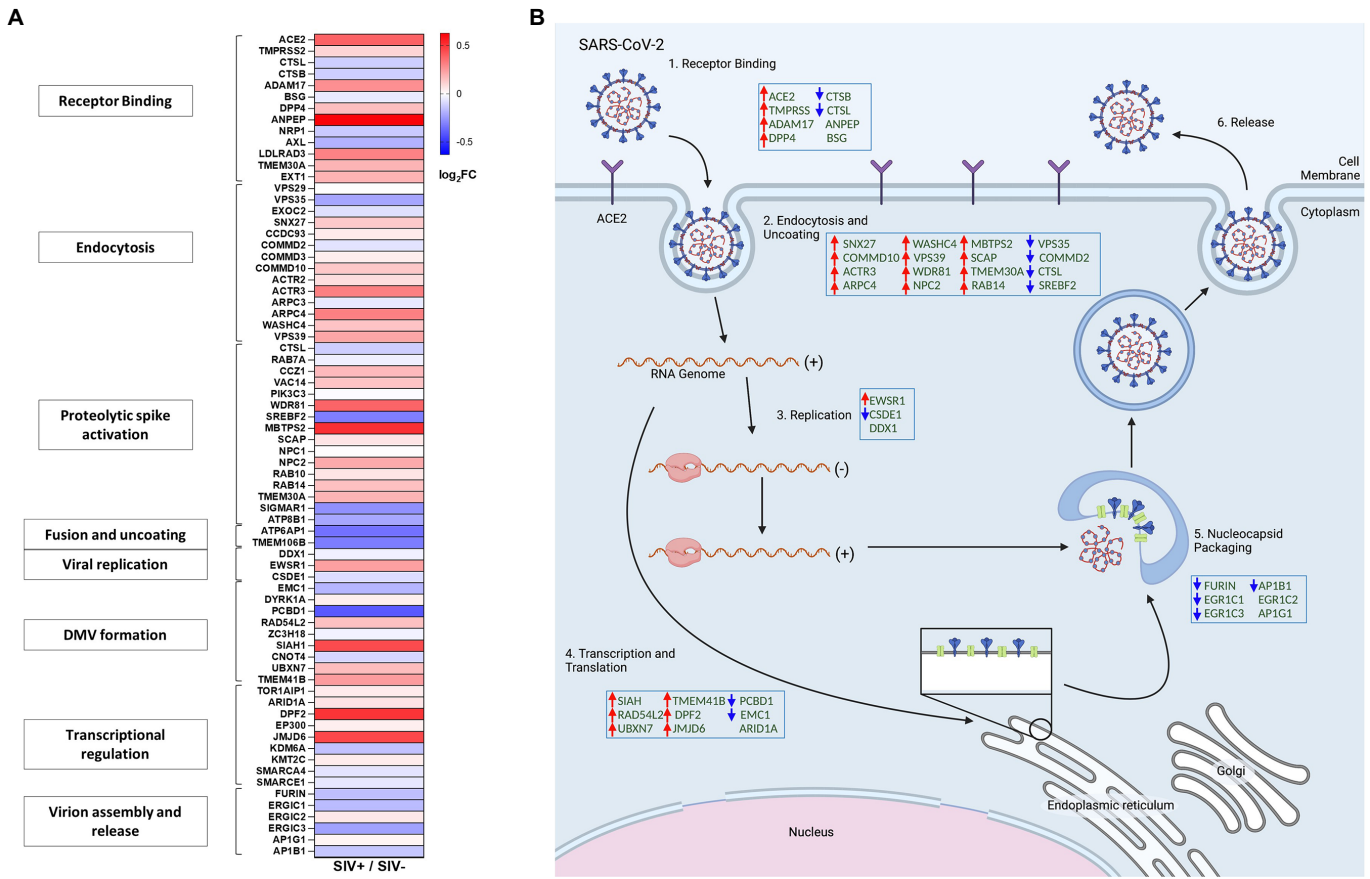
SIV infection modulated gene expression of host factors associated with SARS-CoV-2 replication in the gut. **(A)** Heatmap displays differential expression of genes associated with SARS-CoV-2 viral activity and replication cycles. **(B)** Simplified diagram of the SARS-CoV-2 replication cycle. Host factors associated with SARS-CoV-2 replication cycle are modulated by SIV infection in the gut compartment.

We sought to examine whether HIV induced mucosal changes and associated gene expression networks had the capacity to impact the SARS-CoV-2 infection and viral replication. Gut mucosal gene expression profiles were analyzed from SIV infected animals compared to uninfected controls by using RNAseq analysis. Genes involved in the regulation of ACE2 expression, SARS-CoV-2 co-receptors and related molecular networks were identified and changes in their expression levels evaluated. Our data demonstrated that SIV infection markedly altered the gut gene expression patterns of cellular host factors that promote SARS-CoV-2 attachment and entry and regulate different stages of SARS-CoV-2 replication cycle. An increased expression was observed for SARS-CoV-2 receptors including ACE2, TMPRSS2, ADAM17 as well as of DPP4 which are known to promote viral attachment and entry into the host cell (Figures 4A,B; Hoffmann et al., 2020; Palau et al., 2020; Strollo and Pozzilli, 2020). However, a minor decrease in the expression of Cathepsin L and Cathepsin B that functionally cleave the SARS-CoV-2 spike protein and enhance viral entry was also observed (Figures 4A,B; Padmanabhan et al., 2020; Yang et al., 2021). An increased expression of host factors regulating the endocytosis and uncoating steps in viral replication was found that are associated with retromer/retriever (VPS39 and VPS35), retrograde transport (SNX27), CCC complex (COMMD10 and COMMD2), actin polymerization (ACTR3 and ARPC-4 and WASHC4), PI3K signaling (WDR81), cholesterol homeostasis (NPC2 and MBTPS2), and lipid transport (TMEM30A; Figures 4A,B; Baggen et al., 2021). An increase in the transcript levels was detected for cellular host factors associated with viral transcription and translation (SIAH, RAD54L2, UBXN7, TMEM41B, DPF2, and JMJD6) while, expression of some genes was decreased (ARID1A, EMC1, and PCBD1; Figures 4A,B). Interestingly, host genes associated with SARS-CoV-2 virion assembly and release were mostly downregulated during SIV infection, which include FURIN, ERGIC1, ERGIC3, and AP1B1, while ERGIC2 and AP1G1 expression levels remained relatively the same (Figures 4A,B). Overall, our data showed that the gut mucosal gene expression of cellular host factors associated with receptor binding, viral endocytosis, transcription and translation was generally upregulated in SIV infection.

### Mucosal Changes in Amino Acid and Lipid Metabolism in SIV Infection

**Figure 5:**
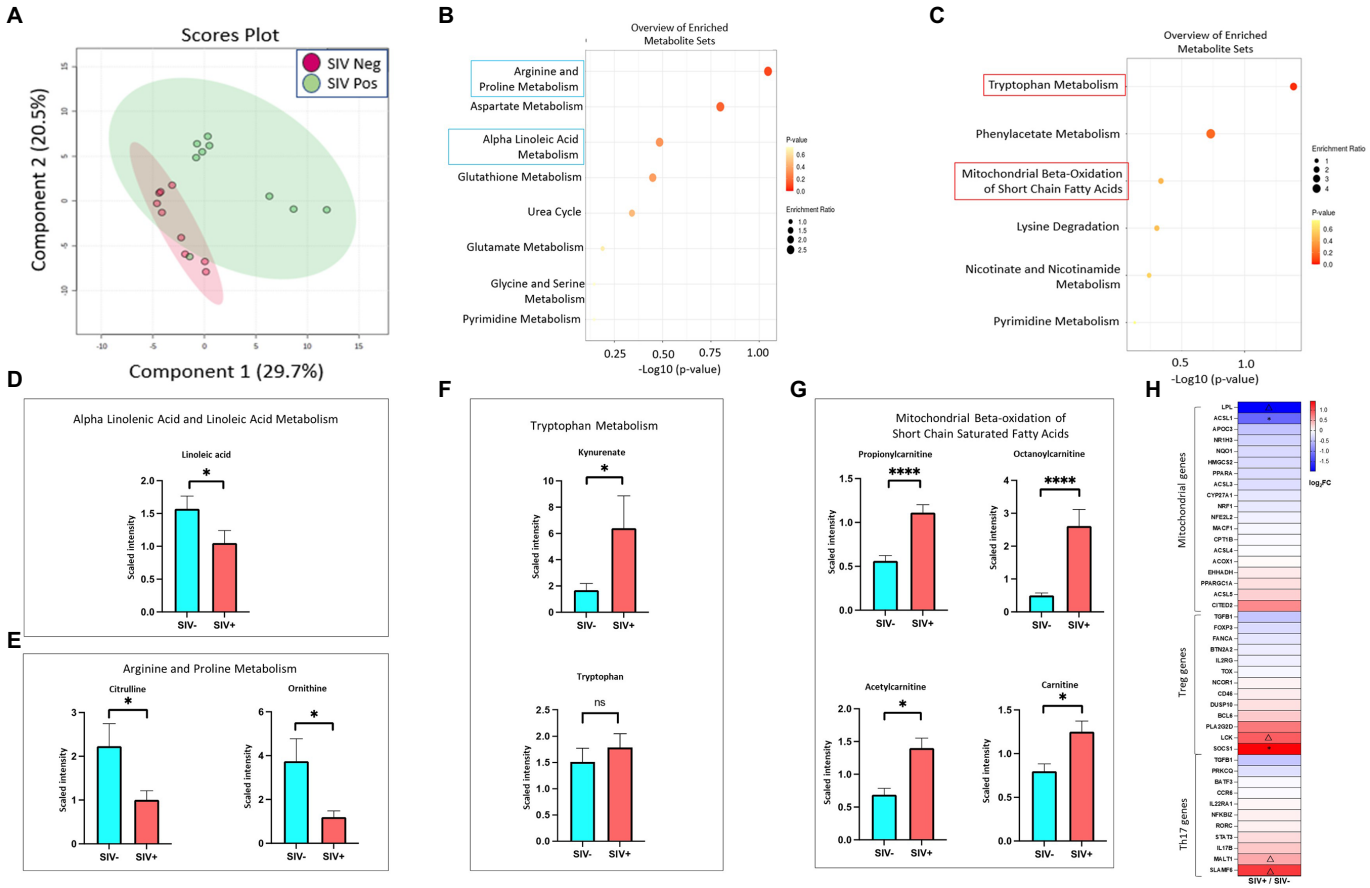
Metabolic changes in the gut reflect persistent ACE2 expression, mitochondrial dysfunction and impaired immune response in SIV infection. **(A)** PLS-DA plots showing separation between groups (SIV-, *n* = 8; SIV+, *n* = 10, 7–10 weeks post infection). Altered metabolites in SIV+ intestinal compartment compared with SIV- controls demonstrated the top 25 downregulated **(B)** and upregulated **(C)** metabolic pathways. Fold changes of linoleic acid **(D)**, citrulline, and ornithine **(E)** within ileum lumen. Fold changes of significantly altered metabolites in the tryptophan metabolism **(F)** and mitochondrial beta-oxidation of short chain fatty acids **(G)**. **(H)** Heatmap displays differential expression of genes associated with mitochondrial function, Treg and Th17 regulations. Δ*p* < 0.1, *p < 0.05, ****p < 0.0001.

**Figure 6:**
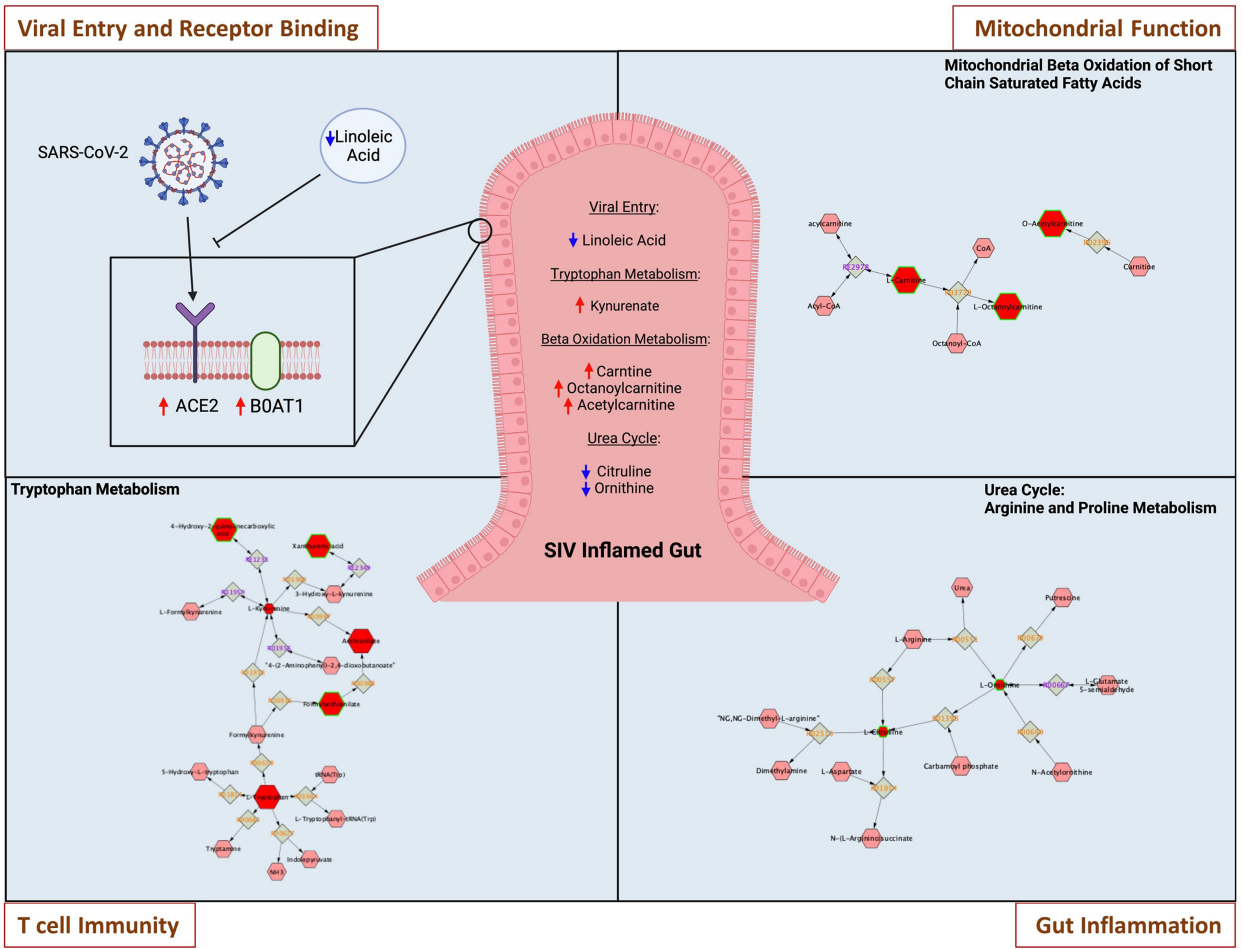
Metabolic changes in the gut during SIV infection increase vulnerability to SARS-CoV-2 infection. Representative networks of metabolites in key pathways are highlighted. SIV induced metabolic changes within inflamed gut directly impact on SARS-CoV-2 viral entry and receptor binding through modulations of linoleic acid and tryptophan transporter-B0AT1. Metabolic pathway of mitochondrial beta oxidation of short chain saturated fatty acids is altered in SIV inflamed gut, leading to mitochondrial malfunction. Tryptophan metabolism is modulated to impair T cell immunity, and dysregulated urea cycle of arginine and proline metabolism leads to intestinal inflammatory stress.

We sought to determine whether SIV induced metabolic changes in the gut could accentuate SARS-CoV-2 related metabolic complications and COVID-19 disease severity. We analyzed metabolomic profiles of the gut luminal contents of SIV infected animals in comparison to uninfected controls. Using all data generated from untargeted metabolomic profiling of luminal contents, we performed partial least-squares discriminant analysis (PLS-DA) to determine separation between SIV infected and uninfected animals. This analysis revealed marked changes in gut metabolism as a consequence of SIV infection (Figure 5A). We identified key metabolites underlying metabolic dysregulation in SIV infection that have relevance to the mucosal immune response (Supplementary Table1). The most pronounced increase was associated with networks regulating tryptophan metabolism and mitochondrial beta-oxidation of short chain saturated fatty acids, while pathways of arginine and proline metabolism and alpha linolenic acid and linoleic acid metabolism were downregulated in SIV infection (Figures 5B,C). We found that alpha linolenic acid and linoleic acid metabolism was significantly altered in SIV infection, and LA levels were decreased in SIV infection (Figure 5D). This can facilitate increased interactions between the viral spike protein and ACE2 receptor and enhance viral entry (Figure 6). Lipid metabolic dysregulation in SIV infection was evident through increased levels of metabolites including propionylcarnitine, octanoylcarnitine, acetylcarnitine, and carnitine (Figures 5G, 6). No changes were observed in downstream metabolites of the TCA cycle, such as malate, fumarate, and succinate (Supplementary Table 1). Transcriptomic analysis in our study found that expression of 15 out of 20 genes related to mitochondrial function was markedly decreased (Figure 5H). SIV infection in the gut resulted in increased levels of tryptophan catabolism as evidenced by the increased levels of kynurenate (Figure 5F). There was no marked change in the levels of tryptophan in the luminal contents of SIV infected animals as compared to the uninfected controls (Figure 5F). These findings suggest that tryptophan absorption and availability was not compromised during SIV infection. The gut mucosal gene expression analysis showed a trend of increased transcription of the broad neutral amino acid transporter (B0AT1) in the gut. These findings suggest that tryptophan uptake mechanism is not impaired in the gut during SIV infection. As previously reported, we found increased tryptophan metabolism in the gut during SIV infection which has direct implication in the host immune response (Crakes et al., 2019). This change was evident by the changes in the expression of genes which regulate Th17 and Treg CD4+ T cell differentiation (Figure 5H). Overall, SIV infection-induced metabolic changes in the gut have impact on the mitochondrial dysfunction and immune dysregulation and increase the vulnerability of the gut microenvironment to SARS-CoV-2 infection and disease severity.

## Discussion

SARS-CoV-2 infection has caused devastating loss of human lives globally. The viral infection causes cytopathic effects on the infected cells and on the neighboring cells through cytokine storm (Coperchini et al., 2020; Yang et al., 2021). There is an urgent need to understand the role of pre-existing infections or co-infections, specifically the mucosal-transmitted viral pathogens, on the clinical outcomes of SARS-CoV-2 infection (Vickers et al., 2002; Neurath, 2020; Alosaimi et al., 2021; Bai et al., 2021; Guo et al., 2021; Yang et al., 2021). Similar to SARS-CoV-2, the majority of HIV infections are spread through mucosal transmission (Hladik and McElrath, 2008). The gastrointestinal tract is an early target organ of HIV infection and a site of massive viral replication that leads to severe CD4+ T cell depletion and epithelial barrier disruption in the gut (Brenchley and Douek, 2008). HIV associated enteropathy impairs mucosal immunity against other pathogens and causes microbial translocation and systemic immune activation (Marchetti et al., 2013). The start of ART suppresses viral burden and restores immune functions in HIV infected people. However, ART is unable to induce complete viral suppression in a large number of HIV-positive individuals which results in incomplete immune recovery and a substantial delay in the repair of the gut mucosal damage (Gaardbo et al., 2012; Reeves et al., 2018). The residual viral replication continues to exert pathogenic effects in the host which could lead to an increased level of susceptibility for SARS-CoV-2 infection. Using the SIV model of AIDS, we examined the impact of SIV infection on the gut mucosal expression of SARS-CoV-2 receptors and host factors involved in the viral replication and virion assembly. This approach enabled us to determine the impact of SIV infection independent of ART effects. ACE2 is highly expressed in the gut epithelium and is critical for SARS-CoV-2 infection (Penninger et al., 2021). However, it is not known whether ACE2 expression is altered in the inflamed gut during untreated HIV infection which could also mimic a suboptimal outcome of ART. We analyzed expression and localization of ACE2 in gut tissues of SIV infected rhesus macaques in comparison to uninfected healthy controls and found that ACE2 expression was well maintained in SIV-inflamed gut compared to uninfected controls. SIV infection is known to cause disruption of the gut epithelial barrier integrity and function and to impair mucosal immunity (Crakes et al., 2019). Thus, maintenance of ACE2 expression in immune compromised gut microenvironment is conducive to SARS-CoV-2 infection and dissemination. It was of interest that the gene expression in SIV infected gut was markedly upregulated for several host factors directly associated with SARS-CoV-2 viral binding, including TMPRSS2, ADAM17, and DPP4. Our findings suggest that pre-existing changes in the gut due to HIV/SIV infection can increase susceptibility to SARS-CoV-2 infection and severity of disease.

Immune dysfunction during HIV infection may provide greater susceptibility to SARS-CoV-2 infection and support the generation of viral variants with genomic mutations and deletions. Recent emergence of the new SARS-CoV-2 variant, B1.1.529 or Omicron, was detected in South Africa and is responsible for the most recent wave of SARS-CoV-2 infections globally. The Omicron variant was thought to originate from an HIV infected individual, which raises concerns about the impact of SARS-CoV-2 and HIV coinfections on the emergence of new viral variants (Freer and Mudaly, 2022). This new SARS-CoV-2 strain has by far the greatest number of mutations (more than 50), with 30 amino acid changes in the spike (S) protein alone (Kumar et al., 2022). These changes may lead to greater susceptibility to SARS-CoV-2 infection and immune escape in vaccinated individuals. Pre-existing immune dysfunction during HIV infection may promote greater susceptibility to SARS-CoV-2 infection and support viral replication in the gut compartment, leading to the generation of viral variants with genomic mutations.

The rhesus macaque model of SARS-CoV-2 infection has been established and used for prevention and therapeutic research (McMahan et al., 2021; Salguero et al., 2021) and it provides a unique opportunity to study the generation, survival, and spread of the SARS-CoV-2 virus in a microenvironment impacted by a pre-existing SIV infection. Experimental SARS-CoV-2 infection in this model often results in mild clinical symptoms which allow for an understanding of how the asymptomatic disease manifests in humans (Chandrashekar et al., 2020; Munster et al., 2020; McMahan et al., 2021; Salguero et al., 2021). Viral RNA and/or antigens were detected in nasal and throat swabs, bronchoalveolar lavage, rectal swabs and gut tissue of infected animals (Munster et al., 2020; Salguero et al., 2021). We analyzed expression and localization of ACE2 in gut tissues from SARS-CoV-2 infected rhesus macaques and found maintenance of robust ACE2 expression in the gut epithelium. This has implication of sustaining viral infection and replication in the gut mucosal tissue.

Previous metabolomic analyses in COVID-19 patients and HIV infected individuals identified signatures of host metabolic abnormalities and physiological dysfunction (Ahmed et al., 2018; Casari et al., 2021). The metabolomic data from the SIV infected gut luminal contents in our study revealed metabolic dysregulation that could prime the gut for SARS-CoV-2 binding to ACE2 receptors and reduce the host anti-viral response. Linoleic acids (LAs) are known to fill alternative binding pockets in the receptor binding domain of the SARS-CoV-2 spike protein, resulting in a decreased affinity between the S protein and ACE2 host receptor (Toelzer et al., 2020). We found that the LA metabolic pathway and LA levels were downregulated during SIV infection which may increase the likelihood of SARS-CoV-2 entry through binding to ACE2 receptor (Figures 5B,D, 6).

Tryptophan metabolism is altered in HIV and SIV infections (Keegan et al., 2016). Tryptophan absorption and metabolism is also modulated in COVID-19 infection, and serves as a marker of long-term clinical complications (Eroğlu et al., 2021). As the main precursor of serotonin and other neurotransmitters, tryptophan is predominantly derived from the diet and absorbed through the intestinal epithelium *via* the amino acid transporter B0AT1 (Gao et al., 2019; Eroğlu et al., 2021). Our data showed an upregulation of tryptophan catabolism as well as increased expression of B0AT1 in the SIV inflamed gut. Since B0AT1 requires formation of a heterodimer with ACE2 to be stable, similar or increased levels of ACE2 expression in the gut epithelium would support the uptake of tryptophan. Increased catabolism of tryptophan to metabolites kynurenine and melatonin have the capacity to modulate the immune system and influence inflammation in infectious diseases by altering the dynamic in Th17/Treg axis (Yan et al., 2010; Hu et al., 2020; Haq et al., 2021). We found a substantial increase in the levels of tryptophan catabolites, including kynurenate which is directly synthesized from kynurenine (Carpenedo et al., 1994; Figures 5C,F). These data suggested that tryptophan and kynurenine in the gut mucosa were rapidly metabolized and converted to downstream catabolic products. Additionally, the transcriptomic data confirmed altered Th17/Treg differentiation and immune activation by SIV infection (Figure 5H). Moreover, mitochondrial functionality was markedly attenuated in the SIV infected gut, as indicated by the altered concentrations of metabolites involved in beta-oxidation of fatty acids. Disruption of this pathway affects mitochondrial respiratory capacity which has significant downstream effects on host physiology and disease (Bjørndal et al., 2018). We found an increase in the levels of short and medium chain acyl-carnitines including propionylcarnitine (2-fold relative to SIV- controls) and octanoylcarnitine (Figure 5G). Our data suggests that the cellular ability to process fatty acids is impaired, leading to an aberrant production chain of electron donating molecules and a consequent decline in ATP production (Silao et al., 2019). Additionally, our data demonstrated that arginine and proline metabolism represented a substantially downregulated pathway in SIV infection. Interestingly, citrulline, a marker of gut immune health, is one of the crucial metabolites in this pathway with a significantly decreased level compared to uninfected controls (Figure 5E; Uyanga et al., 2021). Citrulline acts as a precursor of nitric oxide (NO), which is known to promote gastric epithelial integrity, dampen the local inflammatory response and mitigate severity of mitochondrial disorders (Wallace and Miller, 2000; Kaore et al., 2013; El-Hattab et al., 2014). Overall, our data revealed that the metabolomic landscape associated with SIV infection predisposes the intestinal environment to severe SARS-CoV-2 infection by altering pathways which support mitochondrial health and immune function.

ACE2 expression in the gut is modulated in inflammatory disorders including inflammatory bowel disease (IBD), Crohn’s disease (CD) and ulcerative colitis. Several studies found that ACE2 expression is higher in terminal ileum and colon of inflamed intestine of individuals with IBD or CD (Vickers et al., 2002; Neurath, 2020; Guo et al., 2021; Potdar et al., 2021). The immune mechanism of gut inflammation in the models of IBD and CD involves an increased prevalence of CD4+ Th17 T cells as an inflammatory component (Valverde-Villegas et al., 2015; Smids et al., 2017; Imam et al., 2018). In contrast, severe depletion of CD4+ Th17 cells in the gut occurs very early in HIV and SIV infections (Guadalupe et al., 2003). Effective CD4 + T-cell restoration in gut-associated lymphoid tissue of HIV-infected patients receiving ART is associated with enhanced CD4 + Th17 cells and polyfunctional HIV-specific T-cell responses (Macal et al., 2008). Although ART is effective in suppressing HIV replication, it is unable to directly repair the gut mucosal damage. The recovery of the gut structure and function as well as mucosal immunity in HIV infection is variable and often not complete, even during ART. This leads to increased vulnerability to new infections and associated complications. Therefore, it is important to understand the implications of pre-existing HIV infection in the gut for incoming SARS-CoV-2 infection. Several studies have identified that co-infection with SARS-CoV-2 and influenza A led to increased pathological outcomes in animal models and human patients (Alosaimi et al., 2021; Yang et al., 2021; Zhao et al., 2021). Increased expression of receptors for SARS-CoV-2, ACE2 and TMPRSS2, in human neuronal cells and microglia is reported in HIV infection (Torices et al., 2021). Our study demonstrated a trend of enhanced expression of ACE2 in the gut epithelial lining in the villi during SIV infection *in vivo*, resulting in availability of abundant binding sites for SARS-CoV-2. Moreover, our data also revealed that host factors associated with SARS-CoV-2 replication were upregulated by SIV infection, making the gut prone to new infection of SARS-CoV-2.

In summary, our study reports that abundant ACE2 expression was maintained in gut tissues of SIV infected rhesus macaques in comparison to uninfected controls. Increased transcription of host factors associated with SARS-CoV-2 infection was detected in SIV inflamed gut, implicating increased susceptibility to SARS-CoV-2 infection. Metabolic profiling on gut luminal contents identified changes in the metabolic pathways and metabolites that could lead to persistent expression of ACE2, mitochondrial dysfunction and suboptimal host antiviral immune response. Collectively, our data show that SIV infection led to changes in the gut mucosa which increase the host susceptibility to SARS-CoV-2 and impair generation of effective anti-viral immunity, posing a potential threat for increased severity of disease outcomes.

## Data Availability Statement

The datasets presented in this study can be found in online repositories. The names of the repository/repositories and accession number can be found at: NCBI gene expression omnibus—GSE171635.

## Ethics Statement

The animal study was reviewed and approved by the American Association for Accreditation of Laboratory Animal Care guidelines and Animal Welfare Act/Guide.

## Author Contributions

SD provided conceptual framework. SD and SH developed the experimental design and prepared the manuscript. SH, EB, JA, DR, and CSR performed experiments and data analysis. All authors contributed to the article and approved the submitted version.

## Funding

This work was supported by the NIH grants R01 AI 123105, R37 AI 153025, and P51 OD011107.

## Conflict of Interest

The authors declare that the research was conducted in the absence of any commercial or financial relationships that could be construed as a potential conflict of interest.

## Publisher’s Note

All claims expressed in this article are solely those of the authors and do not necessarily represent those of their affiliated organizations, or those of the publisher, the editors and the reviewers. Any product that may be evaluated in this article, or claim that may be made by its manufacturer, is not guaranteed or endorsed by the publisher.

## Supplementary Material

Supplementary Table 1 Significantly altered metabolites in the luminal contents of SIV+ rhesus macaques compared to SIV- controls.

## References

[ref1] Ablan S. Rawat S. S. Viard M. Wang J. M. Puri A. Blumenthal R. (2006). The role of cholesterol and sphingolipids in chemokine receptor function and HIV-1 envelope glycoprotein-mediated fusion. Virol. J. 3:104. doi: 10.1186/1743-422X-3-104, PMID: 17187670 PMC1769366

[ref2] Ahmed D. Roy D. Cassol E. (2018). Examining relationships between metabolism and persistent inflammation in HIV patients on antiretroviral therapy. Mediat. Inflamm. 2018:6238978. doi: 10.1155/2018/6238978 PMC6181007 30363715

[ref3] Al-Harthi L. Campbell E. Schneider J. A. Bennett D. A. (2021). What HIV in the brain can teach us About SARS-CoV-2 neurological complications? AIDS Res. Hum. Retrovir. 37, 255–265. doi: 10.1089/aid.2020.0161, PMID: 32683890 PMC8035916

[ref4] Alosaimi B. Naeem A. Hamed M. E. Alkadi H. S. Alanazi T. Al Rehily S. S.. (2021). Influenza co-infection associated with severity and mortality in COVID-19 patients. Virol. J. 18:127. doi: 10.1186/s12985-021-01594-0, PMID: 34127006 PMC8200793

[ref5] Ambrosioni J. Blanco J. L. Reyes-Urueña J. M. Davies M. A. Sued O. Marcos M. A.. (2021). Overview of SARS-CoV-2 infection in adults living with HIV. Lancet HIV 8, e294–e305. doi: 10.1016/S2352-3018(21)00070-9, PMID: 33915101 PMC8075775

[ref6] Apicella M. Campopiano M. C. Mantuano M. Mazoni L. Coppelli A. Del Prato S. (2020). COVID-19 in people with diabetes: understanding the reasons for worse outcomes. Lancet Diabetes Endocrinol. 8, 782–792. doi: 10.1016/S2213-8587(20)30238-2, PMID: 32687793 PMC7367664

[ref7] Baggen J. Vanstreels E. Jansen S. Daelemans D. (2021). Cellular host factors for SARS-CoV-2 infection. Nat. Microbiol. 6, 1219–1232. doi: 10.1038/s41564-021-00958-0 34471255

[ref8] Bai L. Zhao Y. Dong J. Liang S. Guo M. Liu X.. (2021). Coinfection with influenza A virus enhances SARS-CoV-2 infectivity. Cell Res. 31, 395–403. doi: 10.1038/s41422-021-00473-1, PMID: 33603116 PMC7890106

[ref9] Bjørndal B. Alterås E. K. Lindquist C. Svardal A. Skorve J. Berge R. K. (2018). Associations between fatty acid oxidation, hepatic mitochondrial function, and plasma acylcarnitine levels in mice. Nutr. Metab. 15:10. doi: 10.1186/s12986-018-0241-7, PMID: 29422939 PMC5789604

[ref10] Brenchley J. M. Douek D. C. (2008). HIV infection and the gastrointestinal immune system. Mucosal Immunol. 1, 23–30. doi: 10.1038/mi.2007.1, PMID: 19079157 PMC2777614

[ref11] Caillet M. Janvier K. Pelchen-Matthews A. Delcroix-Genête D. Camus G. Marsh M.. (2011). Rab7A is required for efficient production of infectious HIV-1. PLoS Pathog. 7:e1002347. doi: 10.1371/journal.ppat.1002347, PMID: 22072966 PMC3207927

[ref12] Carpenedo R. Chiarugi A. Russi P. Lombardi G. Carlà V. Pellicciari R.. (1994). Inhibitors of kynurenine hydroxylase and kynureninase increase cerebral formation of kynurenate and have sedative and anticonvulsant activities. Neuroscience 61, 237–244. doi: 10.1016/0306-4522(94)90227-5, PMID: 7969905

[ref13] Casari I. Manfredi M. Metharom P. Falasca M. (2021). Dissecting lipid metabolism alterations in SARS-CoV-2. Prog. Lipid Res. 82:101092. doi: 10.1016/j.plipres.2021.101092, PMID: 33571544 PMC7869689

[ref14] Chandrashekar A. Liu J. Martinot A. J. McMahan K. Mercado N. B. Peter L.. (2020). SARS-CoV-2 infection protects against rechallenge in rhesus macaques. Science 369, 812–817. doi: 10.1126/science.abc4776, PMID: 32434946 PMC7243369

[ref15] Coperchini F. Chiovato L. Croce L. Magri F. Rotondi M. (2020). The cytokine storm in COVID-19: An overview of the involvement of the chemokine/chemokine-receptor system. Cytokine Growth Factor Rev. 53, 25–32. doi: 10.1016/j.cytogfr.2020.05.003, PMID: 32446778 PMC7211650

[ref16] Crakes K. R. Santos Rocha C. Grishina I. Hirao L. A. Napoli E. Gaulke C. A.. (2019). PPARα-targeted mitochondrial bioenergetics mediate repair of intestinal barriers at the host–microbe intersection during SIV infection. Proc. Natl. Acad. Sci. 116, 24819–24829. doi: 10.1073/pnas.1908977116, PMID: 31740620 PMC6900595

[ref17] Dai L. Gao G. F. (2021). Viral targets for vaccines against COVID-19. Nat. Rev. Immunol. 21, 73–82. doi: 10.1038/s41577-020-00480-0, PMID: 33340022 PMC7747004

[ref18] Daniloski Z. Jordan T. X. Wessels H. H. Hoagland D. A. Kasela S. Legut M.. (2021). Identification of required host factors for SARS-CoV-2 infection in human cells. Cell 184, 92–105. doi: 10.1016/j.cell.2020.10.030, PMID: 33147445 PMC7584921

[ref19] Dauby N. Martin C. (2021). SARS-CoV-2 immunity and HIV infection: total recall? Lancet HIV 8, e312–e313. doi: 10.1016/S2352-3018(21)00097-7, PMID: 33933190 PMC8084353

[ref20] Devaux C. A. Rolain J. M. Raoult D. (2020). ACE2 receptor polymorphism: susceptibility to SARS-CoV-2, hypertension, multi-organ failure, and COVID-19 disease outcome. J. Microbiol. Immunol. Infect. 53, 425–435. doi: 10.1016/j.jmii.2020.04.015, PMID: 32414646 PMC7201239

[ref21] El-Hattab A. W. Emrick L. T. Chanprasert S. Craigen W. J. Scaglia F. (2014). Mitochondria: role of citrulline and arginine supplementation in MELAS syndrome. Int. J. Biochem. Cell Biol. 48, 85–91. doi: 10.1016/j.biocel.2013.12.009, PMID: 24412347

[ref22] Eroğlu İ. Eroğlu B. Güven G. S. (2021). Altered tryptophan absorption and metabolism could underlie long-term symptoms in survivors of coronavirus disease 2019 (COVID-19). Nutrition 90:111308. doi: 10.1016/j.nut.2021.111308, PMID: 34111831 PMC8087860

[ref23] Fardoos R. Asowata O. E. Herbert N. Nyquist S. K. Zungu Y. Singh A.. (2021). HIV infection drives interferon signaling within intestinal SARS-CoV-2 target cells. JCI Insight 6:148920. doi: 10.1172/jci.insight.148920, PMID: 34252054 PMC8409978

[ref24] Freer J. Mudaly V. (2022). HIV and covid-19 in South Africa. BMJ 376:e069807. doi: 10.1136/bmj-2021-069807 35086921

[ref25] Gaardbo J. C. Hartling H. J. Gerstoft J. Nielsen S. D. (2012). Incomplete immune recovery in HIV infection: mechanisms, relevance for clinical care, and possible solutions. Clin. Dev. Immunol. 2012:670957. doi: 10.1155/2012/670957 22474480 PMC3312328

[ref26] Gao K. Mu C.-L. Farzi A. Zhu W.-Y. (2019). Tryptophan metabolism: a link between the gut microbiota and brain. Adv. Nutr. 11, 709–723. doi: 10.1093/advances/nmz127 PMC7231603 31825083

[ref27] Gostner J. M. Becker K. Kurz K. Fuchs D. (2015). Disturbed amino acid metabolism in HIV: association with neuropsychiatric symptoms. Front. Psych. 6:97. doi: 10.3389/fpsyt.2015.00097 PMC4500866 26236243

[ref28] Guadalupe M. Reay E. Sankaran S. Prindiville T. Flamm J. McNeil A.. (2003). Severe CD4+ T-cell depletion in gut lymphoid tissue during primary human immunodeficiency virus type 1 infection and substantial delay in restoration following highly active antiretroviral therapy. J. Virol. 77, 11708–11717. doi: 10.1128/JVI.77.21.11708-11717.2003, PMID: 14557656 PMC229357

[ref29] Guo Y. Wang B. Gao H. Gao L. Hua R. Xu J.-D. (2021). ACE2 in the gut: The center of the 2019-nCoV infected pathology. Front. Mol. Biosci. 8:708336. doi: 10.3389/fmolb.2021.708336, PMID: 34631794 PMC8493804

[ref30] Haq S. Grondin J. A. Khan W. I. (2021). Tryptophan-derived serotonin-kynurenine balance in immune activation and intestinal inflammation. FASEB J. 35:e21888. doi: 10.1096/fj.202100702R, PMID: 34473368 PMC9292703

[ref31] Hirao L. A. Grishina I. Bourry O. Hu W. K. Somrit M. Sankaran-Walters S.. (2014). Early mucosal sensing of SIV infection by paneth cells induces IL-1β production and initiates gut epithelial disruption. PLoS Pathog. 10:e1004311. doi: 10.1371/journal.ppat.1004311, PMID: 25166758 PMC4148401

[ref32] Hladik F. McElrath M. J. (2008). Setting the stage: host invasion by HIV. Nat. Rev. Immunol. 8, 447–457. doi: 10.1038/nri2302, PMID: 18469831 PMC2587276

[ref33] Hoffmann M. Kleine-Weber H. Schroeder S. Krüger N. Herrler T. Erichsen S.. (2020). SARS-CoV-2 cell entry depends on ACE2 and TMPRSS2 and is blocked by a clinically proven protease inhibitor. Cell 181, 271–280.e8. doi: 10.1016/j.cell.2020.02.052, PMID: 32142651 PMC7102627

[ref34] Hu B. Guo H. Zhou P. Shi Z.-L. (2021). Characteristics of SARS-CoV-2 and COVID-19. Nat. Rev. Microbiol. 19, 141–154. doi: 10.1038/s41579-020-00459-7, PMID: 33024307 PMC7537588

[ref35] Hu Q. Jin L. Zeng J. Wang J. Zhong S. Fan W.. (2020). Tryptophan metabolite-regulated Treg responses contribute to attenuation of airway inflammation during specific immunotherapy in a mouse asthma model. Hum. Vaccin. Immunother. 16, 1891–1899. doi: 10.1080/21645515.2019.1698900, PMID: 31951781 PMC7482871

[ref36] Hu S. McCartney M. M. Arredondo J. Sankaran-Walters S. Borras E. Harper R. W.. (2021). Inactivation of SARS-CoV-2 in clinical exhaled breath condensate samples for metabolomic analysis. J. Breath Res. 16. doi: 10.1088/1752-7163/ac3f24 PMC9809239 34852327

[ref37] Imam T. Park S. Kaplan M. H. Olson M. R. (2018). Effector T helper cell subsets in inflammatory bowel diseases. Front. Immunol. 9:1212. doi: 10.3389/fimmu.2018.01212, PMID: 29910812 PMC5992276

[ref38] Kanwugu O. N. Adadi P. (2021). HIV/SARS-CoV-2 coinfection: A global perspective. J. Med. Virol. 93, 726–732. doi: 10.1002/jmv.26321, PMID: 32692406 PMC7404432

[ref39] Kaore S. N. Amane H. S. Kaore N. M. (2013). Citrulline: pharmacological perspectives and its role as an emerging biomarker in future. Fundam. Clin. Pharmacol. 27, 35–50. doi: 10.1111/j.1472-8206.2012.01059.x, PMID: 23316808

[ref40] Keegan M. R. Chittiprol S. Letendre S. L. Winston A. Fuchs D. Boasso A.. (2016). Tryptophan metabolism and its relationship with depression and cognitive impairment Among HIV-infected individuals. Int. J. Tryptophan Res. 9, 79–88. doi: 10.4137/IJTR.S36464 27812290 PMC5083113

[ref41] Krammer F. (2020). SARS-CoV-2 vaccines in development. Nature 586, 516–527. doi: 10.1038/s41586-020-2798-3 32967006

[ref42] Kumar S. Thambiraja T. S. Karuppanan K. Subramaniam G. (2022). Omicron and Delta variant of SARS-CoV-2: A comparative computational study of spike protein. J. Med. Virol. 94, 1641–1649. doi: 10.1002/jmv.27526, PMID: 34914115

[ref43] Lamers M. M. Beumer J. van der Vaart J. Knoops K. Puschhof J. Breugem T. I.. (2020). SARS-CoV-2 productively infects human gut enterocytes. Science 369, 50–54. doi: 10.1126/science.abc1669, PMID: 32358202 PMC7199907

[ref44] Lim S. Bae J. H. Kwon H.-S. Nauck M. A. (2021). COVID-19 and diabetes mellitus: from pathophysiology to clinical management. Nat. Rev. Endocrinol. 17, 11–30. doi: 10.1038/s41574-020-00435-4, PMID: 33188364 PMC7664589

[ref45] Macal M. Sankaran S. Chun T. W. Reay E. Flamm J. Prindiville T. J.. (2008). Effective CD4+ T-cell restoration in gut-associated lymphoid tissue of HIV-infected patients is associated with enhanced Th17 cells and polyfunctional HIV-specific T-cell responses. Mucosal Immunol. 1, 475–488. doi: 10.1038/mi.2008.35, PMID: 19079215

[ref46] Maggi P. Di Biagio A. Rusconi S. Cicalini S. D'Abbraccio M. d'Ettorre G.. (2017). Cardiovascular risk and dyslipidemia among persons living with HIV: a review. BMC Infect. Dis. 17:551. doi: 10.1186/s12879-017-2626-z, PMID: 28793863 PMC5550957

[ref47] Marchetti G. Tincati C. Silvestri G. (2013). Microbial translocation in the pathogenesis of HIV infection and AIDS. Clin. Microbiol. Rev. 26, 2–18. doi: 10.1128/CMR.00050-12, PMID: 23297256 PMC3553668

[ref48] McMahan K. Yu J. Mercado N. B. Loos C. Tostanoski L. H. Chandrashekar A.. (2021). Correlates of protection against SARS-CoV-2 in rhesus macaques. Nature 590, 630–634. doi: 10.1038/s41586-020-03041-6, PMID: 33276369 PMC7906955

[ref49] Mollica V. Rizzo A. Massari F. (2020). The pivotal role of TMPRSS2 in coronavirus disease 2019 and prostate cancer. Future Oncol. 16, 2029–2033. doi: 10.2217/fon-2020-0571, PMID: 32658591 PMC7359420

[ref50] Munster V. J. Feldmann F. Williamson B. N. van Doremalen N. Pérez-Pérez L. Schulz J.. (2020). Respiratory disease in rhesus macaques inoculated with SARS-CoV-2. Nature 585, 268–272. doi: 10.1038/s41586-020-2324-7, PMID: 32396922 PMC7486227

[ref51] Neurath M. F. (2020). COVID-19 and immunomodulation in IBD. Gut 69, 1335–1342. doi: 10.1136/gutjnl-2020-321269, PMID: 32303609 PMC7211083

[ref52] Padmanabhan P. Desikan R. Dixit N. M. (2020). Targeting TMPRSS2 and Cathepsin B/L together may be synergistic against SARS-CoV-2 infection. PLoS Comput. Biol. 16:e1008461. doi: 10.1371/journal.pcbi.1008461, PMID: 33290397 PMC7748278

[ref53] Palau V. Riera M. Soler M. J. (2020). ADAM17 inhibition may exert a protective effect on COVID-19. Nephrol. Dial. Transplant. 35, 1071–1072. doi: 10.1093/ndt/gfaa093, PMID: 32291449 PMC7184459

[ref54] Penninger J. M. Grant M. B. Sung J. J. Y. (2021). The role of angiotensin converting enzyme 2 in modulating gut microbiota, intestinal inflammation, and coronavirus infection. Gastroenterology 160, 39–46. doi: 10.1053/j.gastro.2020.07.067, PMID: 33130103 PMC7836226

[ref55] Potdar A. A. Dube S. Naito T. Li K. Botwin G. Haritunians T.. (2021). Altered intestinal ACE2 levels are associated with inflammation, severe disease, and response to anti-cytokine therapy in inflammatory bowel disease. Gastroenterology 160, 809–822.e7. doi: 10.1053/j.gastro.2020.10.041, PMID: 33160965 PMC9671555

[ref56] Qi M. Williams J. A. Chu H. Chen X. Wang J. J. Ding L.. (2013). Rab11-FIP1C and Rab14 direct plasma membrane sorting and particle incorporation of the HIV-1 envelope glycoprotein complex. PLoS Pathog. 9:e1003278. doi: 10.1371/journal.ppat.1003278, PMID: 23592992 PMC3616983

[ref57] Rasheed S. Yan J. S. Lau A. Chan A. S. (2008). HIV replication enhances production of free fatty acids, low density lipoproteins and many key proteins involved in lipid metabolism: a proteomics study. PLoS One 3:e3003. doi: 10.1371/journal.pone.0003003, PMID: 18714345 PMC2500163

[ref58] Reeves D. B. Duke E. R. Wagner T. A. Palmer S. E. Spivak A. M. Schiffer J. T. (2018). A majority of HIV persistence during antiretroviral therapy is due to infected cell proliferation. Nat. Commun. 9:4811. doi: 10.1038/s41467-018-06843-5, PMID: 30446650 PMC6240116

[ref59] Rosano G. Jankowska E. A. Ray R. Metra M. Abdelhamid M. Adamopoulos S.. (2021). COVID-19 vaccination in patients with heart failure: a position paper of the Heart Failure Association of the European Society of Cardiology. Eur. J. Heart Fail 23, 1806–1818. 34612556 10.1002/ejhf.2356 PMC8652673

[ref60] Salamanna F. Maglio M. Landini M. P. Fini M. (2020). Body localization of ACE-2: On the trail of the keyhole of SARS-CoV-2. Front. Med. 7:594495. doi: 10.3389/fmed.2020.594495, PMID: 33344479 PMC7744810

[ref61] Salguero F. J. White A. D. Slack G. S. Fotheringham S. A. Bewley K. R. Gooch K. E.. (2021). Comparison of rhesus and cynomolgus macaques as an infection model for COVID-19. Nat. Commun. 12:1260. doi: 10.1038/s41467-021-21389-9, PMID: 33627662 PMC7904795

[ref62] Scarpellini B. Zanoni M. Sucupira M. C. Truong H. M. Janini L. M. Segurado I. D.. (2016). Plasma metabolomics biosignature according to HIV stage of infection, pace of disease progression, viremia level and immunological response to treatment. PLoS One 11:e0161920. doi: 10.1371/journal.pone.0161920, PMID: 27941971 PMC5152829

[ref63] Shaan Lakshmanappa Y. Elizaldi S. R. Roh J. W. Schmidt B. A. Carroll T. D. Weaver K. D.. (2021). SARS-CoV-2 induces robust germinal center CD4 T follicular helper cell responses in rhesus macaques. Nat. Commun. 12:541. doi: 10.1038/s41467-020-20642-x, PMID: 33483492 PMC7822826

[ref64] Silao F. G. S. Ward M. Ryman K. Wallström A. Brindefalk B. Udekwu K.. (2019). Mitochondrial proline catabolism activates Ras1/cAMP/PKA-induced filamentation in Candida albicans. PLoS Genet. 15:e1007976. doi: 10.1371/journal.pgen.1007976, PMID: 30742618 PMC6386415

[ref65] Smids C. Horje C. S. H. T. Drylewicz J. Roosenboom B. Groenen M. J. M. van Koolwijk E.. (2017). Intestinal T cell profiling in inflammatory bowel disease: linking T cell subsets to disease activity and disease course. J. Crohn's Colitis 12, 465–475. doi: 10.1093/ecco-jcc/jjx160 29211912

[ref66] Strollo R. Pozzilli P. (2020). DPP4 inhibition: preventing SARS-CoV-2 infection and/or progression of COVID-19? Diabetes Metab. Res. Rev. 36:e3330. doi: 10.1002/dmrr.3330, PMID: 32336007 PMC7267128

[ref67] Suárez-Fariñas M. Tokuyama M. Wei G. Huang R. Livanos A. Jha D.. (2021). Intestinal inflammation modulates the expression of ACE2 and TMPRSS2 and potentially overlaps With the pathogenesis of SARS-CoV-2-related disease. Gastroenterology 160, 287–301. doi: 10.1053/j.gastro.2020.09.029, PMID: 32980345 PMC7516468

[ref68] Suryamohan K. Diwanji D. Stawiski E. W. Gupta R. Miersch S. Liu J.. (2021). Human ACE2 receptor polymorphisms and altered susceptibility to SARS-CoV-2. Commun. Biol. 4:475. doi: 10.1038/s42003-021-02030-3, PMID: 33846513 PMC8041869

[ref69] Tesoriero J. M. Swain C.-A. E. Pierce J. L. Zamboni L. Wu M. Holtgrave D. R.. (2021). COVID-19 outcomes Among persons living With or Without diagnosed HIV infection in New York state. JAMA Netw. Open 4:e2037069. doi: 10.1001/jamanetworkopen.2020.37069, PMID: 33533933 PMC7859843

[ref70] Thompson C. G. Gay C. L. Kashuba A. D. M. (2017). HIV persistence in gut-associated lymphoid tissues: pharmacological challenges and opportunities. AIDS Res. Hum. Retrovir. 33, 513–523. doi: 10.1089/aid.2016.0253, PMID: 28398774 PMC5467125

[ref71] Toelzer C. Gupta K. Yadav S. K. N. Borucu U. Davidson A. D. Kavanagh Williamson M.. (2020). Free fatty acid binding pocket in the locked structure of SARS-CoV-2 spike protein. Science 370, 725–730. doi: 10.1126/science.abd3255, PMID: 32958580 PMC8050947

[ref72] Torices S. Cabrera R. Stangis M. Naranjo O. Fattakhov N. Teglas T.. (2021). Expression of SARS-CoV-2-related receptors in cells of the neurovascular unit: implications for HIV-1 infection. J. Neuroinflammation 18:167. doi: 10.1186/s12974-021-02210-2, PMID: 34325716 PMC8319595

[ref73] Trypsteen W. Van Cleemput J. Snippenberg W. V. Gerlo S. Vandekerckhove L. (2020). On the whereabouts of SARS-CoV-2 in the human body: A systematic review. PLoS Pathog. 16:e1009037. doi: 10.1371/journal.ppat.1009037, PMID: 33125439 PMC7679000

[ref74] Uyanga V. A. Amevor F. K. Liu M. Cui Z. Zhao X. Lin H. (2021). Potential implications of Citrulline and Quercetin on gut functioning of Monogastric animals and humans: a comprehensive review. Nutrients 13:3782. doi: 10.3390/nu13113782, PMID: 34836037 PMC8621968

[ref75] Valverde-Villegas J. M. Matte M. C. C. de Medeiros R. M. Chies J. A. B. (2015). New insights about Treg and Th17 cells in HIV infection and disease progression. J. Immunol. Res. 2015:647916. doi: 10.1155/2015/647916 26568963 PMC4629044

[ref76] Veazey R. S. (2019). Intestinal CD4 depletion in HIV / SIV infection. Curr. Immunol. Rev. 15, 76–91. doi: 10.2174/1573395514666180605083448, PMID: 31431807 PMC6701936

[ref77] Verhoeven D. Sankaran S. Silvey M. Dandekar S. (2008). Antiviral therapy during primary simian immunodeficiency virus infection fails to prevent acute loss of CD4+ T cells in gut mucosa but enhances their rapid restoration through central memory T cells. J. Virol. 82, 4016–4027. doi: 10.1128/JVI.02164-07, PMID: 18272585 PMC2292978

[ref78] Viana S. D. Nunes S. Reis F. (2020). ACE2 imbalance as a key player for the poor outcomes in COVID-19 patients with age-related comorbidities – role of gut microbiota dysbiosis. Ageing Res. Rev. 62:101123. doi: 10.1016/j.arr.2020.101123, PMID: 32683039 PMC7365123

[ref79] Vickers C. Hales P. Kaushik V. Dick L. Gavin J. Tang J.. (2002). Hydrolysis of biological peptides by human angiotensin-converting enzyme-related carboxypeptidase. J. Biol. Chem. 277, 14838–14843. doi: 10.1074/jbc.M200581200, PMID: 11815627

[ref80] Wallace J. L. Miller M. J. (2000). Nitric oxide in mucosal defense: a little goes a long way. Gastroenterology 119, 512–520. doi: 10.1053/gast.2000.9304, PMID: 10930387

[ref81] Wang Y. Liu S. Liu H. Li W. Lin F. Jiang L.. (2020). SARS-CoV-2 infection of the liver directly contributes to hepatic impairment in patients with COVID-19. J. Hepatol. 73, 807–816. doi: 10.1016/j.jhep.2020.05.002, PMID: 32437830 PMC7211738

[ref82] Weber M. G. Walters-Laird C. J. Kol A. Santos Rocha C. Hirao L. A. Mende A.. (2021). "Gut germinal center regeneration and enhanced antiviral immunity by mesenchymal stem/stromal cells in SIV infection." JCI. Insight 6:e149033. doi: 10.1172/jci.insight.149033 PMC8262475 34014838

[ref83] Wölfel R. Corman V. M. Guggemos W. Seilmaier M. Zange S. Müller M. A.. (2020). Virological assessment of hospitalized patients with COVID-2019. Nature 581, 465–469. doi: 10.1038/s41586-020-2196-x, PMID: 32235945

[ref84] Yan Y. Zhang G.-X. Gran B. Fallarino F. Yu S. Li H.. (2010). IDO upregulates regulatory T cells via tryptophan catabolite and suppresses encephalitogenic T cell responses in experimental autoimmune encephalomyelitis. J. Immun. 185, 5953–5961. 20944000 10.4049/jimmunol.1001628 PMC2998795

[ref85] Yang L. Xie X. Tu Z. Fu J. Xu D. Zhou Y. (2021). The signal pathways and treatment of cytokine storm in COVID-19. Signal Transduct. Target. Ther. 6:255. doi: 10.1038/s41392-021-00679-0, PMID: 34234112 PMC8261820

[ref86] Zhang Y. Coats A. J. S. Zheng Z. Adamo M. Ambrosio G. Anker S. D.. (2020). Management of heart failure patients with COVID-19: a joint position paper of the Chinese heart failure Association & National Heart Failure Committee and the heart failure Association of the European Society of cardiology. Eur. J. Heart Fail. 22, 941–956. doi: 10.1002/ejhf.1915, PMID: 32463543

[ref87] Zhang X. Tan Y. Ling Y. Lu G. Liu F. Yi Z.. (2020). Viral and host factors related to the clinical outcome of COVID-19. Nature 583, 437–440. doi: 10.1038/s41586-020-2355-0, PMID: 32434211

[ref88] Zhao M.-M. Yang W.-L. Yang F.-Y. Zhang L. Huang W.-J. Hou W.. (2021). Cathepsin L plays a key role in SARS-CoV-2 infection in humans and humanized mice and is a promising target for new drug development. Signal Transduct. Target. Ther. 6:134. doi: 10.1038/s41392-021-00558-8, PMID: 33774649 PMC7997800

